# Precision and consistency of the passive leg raising maneuver for determining fluid responsiveness with bioreactance non-invasive cardiac output monitoring in critically ill patients and healthy volunteers

**DOI:** 10.1371/journal.pone.0222956

**Published:** 2019-09-27

**Authors:** Sahil Chopra, Jordan Thompson, Shahab Shahangian, Suman Thapamagar, Dafne Moretta, Chris Gasho, Avi Cohen, H. Bryant Nguyen

**Affiliations:** 1 Division of Pulmonary, Critical Care, Hyperbaric, Allergy and Sleep Medicine, Loma Linda University, Loma Linda, California, United States of America; 2 School of Medicine, Loma Linda University, Loma Linda, California, United States of America; 3 Department of Medicine, Loma Linda University, Loma Linda, California, United States of America; Cleveland Clinic, UNITED STATES

## Abstract

**Objective:**

The passive leg raising (PLR) maneuver has become standard practice in fluid resuscitation. We aim to investigate the precision and consistency of the PLR for determining fluid responsiveness in critically ill patients and healthy volunteers using bioreactance non-invasive cardiac output monitoring (NiCOM™, Cheetah Medical, Inc., Newton Center, Massachusetts, USA).

**Methods:**

This study is prospective, single-center, observational cohort with repeated measures in critically ill patients admitted to the medical intensive care unit and healthy volunteers at a tertiary academic medical center. Three cycles of PLR were performed, each at 20–30 minutes apart. *Fluid responsiveness* was defined as a change in stroke volume index (ΔSVI) > 10% with each PLR as determined by NiCOM™. *Precision* was the variability in ΔSVI after the 3 PLR’s, and determined by *range*, *average deviation* and *standard deviation*. *Consistency* was the same fluid responsiveness determination of “Yes” (ΔSVI > 10%) or “No” (ΔSVI ≤ 10%) for all 3 PLR’s.

**Results:**

Seventy-five patients and 25 volunteers were enrolled. In patients, the precision was range of 17.2±13.3%, average deviation 6.5±4.0% and standard deviation 9.0±5.2%; and for volunteers, 17.4±10.3%, 6.6±3.8% and 9.0±6.7%, respectively. There was no statistical difference in the precision measurements between patients and volunteers. Forty-nine (65.3%) patients vs. twenty-four (96.0%) volunteers had consistent results, p < 0.01. Among those with consistent results, twenty-four (49.0%) patients and 24 (100%) volunteers were fluid responsive.

**Conclusions:**

The precision and consistency of determining ΔSVI with NiCOM™ after PLR may have clinical implication if ΔSVI > 10% is the absolute cutoff to determine fluid responsiveness.

## Introduction

Fluid management decisions are a difficult but integral part of daily patient care. With the growing consensus that extremes of fluid balance may be detrimental, an accurate assessment of a patient’s volume status is paramount [1–4]. Screening patients who are fluid responsive can appropriately identify those who would likely benefit from fluid resuscitation.

Fluid responsiveness as an increase in cardiac output (CO) in response to augmentation of preload is fundamentally based on the classical Frank-Starling curve [5, 6]. If preload augmentation does not increase CO, further intravenous fluids serve no purpose and may potentially be harmful. However, clinicians often face the challenge of accurately recognizing when a patient has reached this plateau on the Frank-Starling curve. One maneuver, which has gained acceptance in standard practice to predict fluid responsiveness, without possibly inappropriate fluid administration, is passive leg raising (PLR) [7–9].

With less invasive technologies becoming increasingly available, the gold standard of pulmonary artery (PA) catheterization to measure CO has naturally fallen out of favor. As such, the bioreactance-based Non-invasive Cardiac Output Monitor (NiCOM™, Cheetah Medical, Inc., Newton Center, Massachusetts, USA) became popular in recent years given its completely non-invasive platform using four skin sensor pads placed on the patient’s thorax. It measures stroke volume (SV) by using time delays or phase shifts induced by thoracic blood flow during the transmission of electrical current between the sensors [10–12].

At our institution, the NiCOM™ technology has become a primary tool to guide fluid management. We utilize its built-in algorithm for determining fluid responsiveness with the PLR. We a priori accept the NiCOM™ to provide accurate measurements of SV and CO. However, we frequently observed variability in the measured change in stroke volume index (ΔSVI) based on PLR. While the *accuracy* of the PLR for predicting fluid responsiveness has been determined, we were unable to find published data on the *precision* and *consistency* of predicting fluid responsiveness in critically ill patients by PLR [13]. The purpose of this study was to determine the precision and consistency of the PLR in predicting fluid responsiveness utilizing the NiCOM™ technology in critically ill patients and healthy volunteers.

## Materials and methods

### Study design

This study is a single-center, prospective observational cohort, with repeated measures in critically ill patients admitted to the medical intensive care unit and healthy volunteers. The study was conducted at an academic tertiary-care medical center from June 2017 to January 2018. Written informed consent was obtained from study participants or their legal representatives. The protocol was approved by the Loma Linda University Institutional Review Board.

### Study participants

For the patient cohort, inclusion criteria were: 1) age ≥ 18 years, and 2) suspicion for hypovolemia or indicated for volume expansion due to any one of the following: hypotension (systolic blood pressure < 90 mm Hg or mean arterial pressure < 65 mm Hg), tachycardia (heart rate > 90 beats per min), blood lactate > 2.0 mmol/L, skin mottling, oliguria (urine output < 30 ml/hr), or requiring vasopressor/inotrope support. Patients were excluded if there was an active arrhythmia, bleeding causing hemodynamic instability, intra-abdominal hypertension, pelvic and/or lower extremity trauma/amputation preventing passive leg raising, pulmonary hypertension requiring chronic pulmonary vasodilator therapy, severe valvular heart disease, severe anatomic abnormalities of thoracic aorta, external pacemaker, or renal replacement therapy during the data collection period.

Healthy volunteers were enrolled if they were ≥ 18 years of age with self-reported unremarkable medical history, but then excluded if unable to tolerate PLR.

### Study procedure and data collection

**Fig 1:**
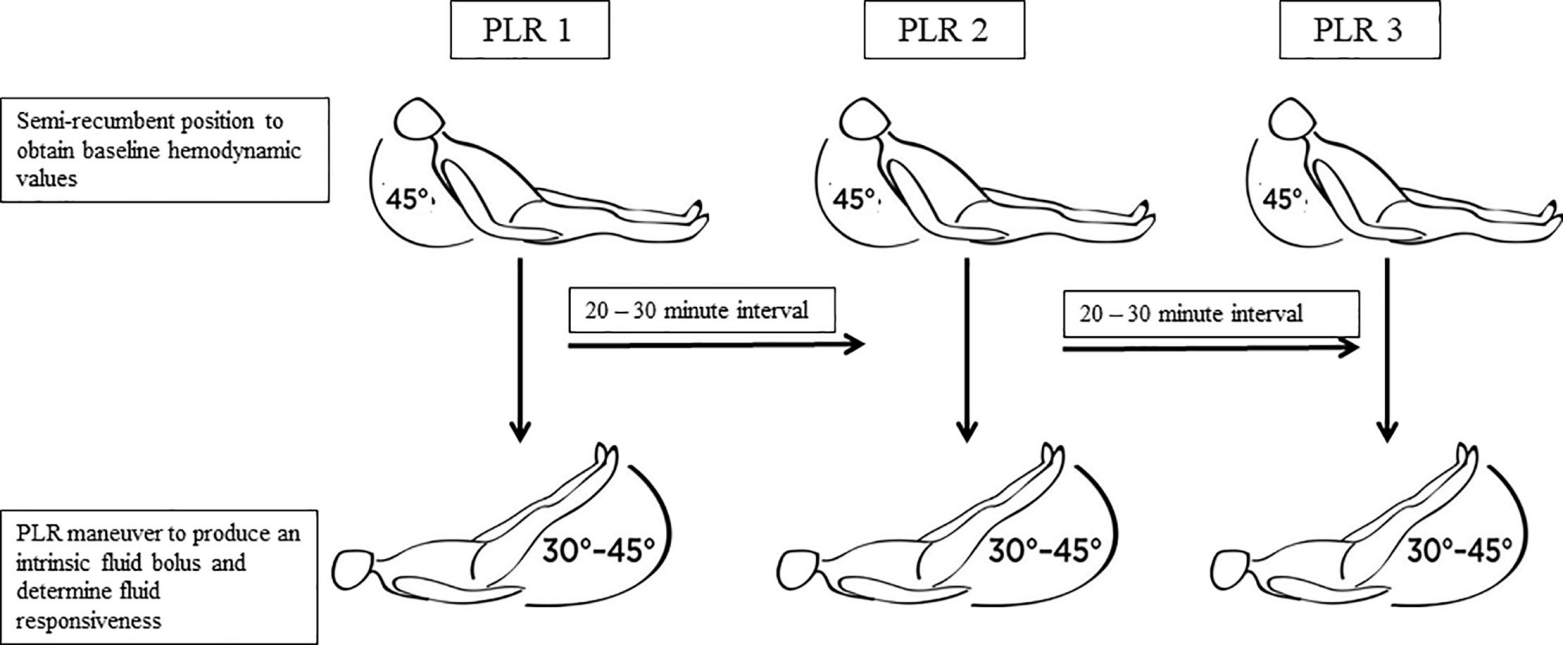
Serial passive leg raising (PLR) maneuver protocol. Patients were placed in the semi-recumbent position at 45° for 3 minutes and baseline hemodynamic values were recorded. PLR was induced by raising both lower extremities to 30–45° using a standard wedge pillow and held in this position for 3 minutes. After data collection, the patient was returned to the semi-recumbent resting position for 20–30 minutes. The PLR maneuver was repeated for 2 additional cycles.

To ensure minimal variability, the PLR and data collection were performed only by the authors after they were provided with an in-depth training on the NiCOM™ technology by the manufacturer. Each PLR and associated hemodynamic parameters were performed and recorded as follows. The subject’s head was raised to a semi-recumbent 45° position for 3 minutes with the legs flat in the bed. After a stable hemodynamic signal was achieved on the NiCOM™ system, baseline hemodynamic data was collected. The subject’s head was then placed flat and the legs elevated to 30–45° for 3 minutes with a standardized wedge pillow. The second set of hemodynamic data was collected at the time of PLR “auto-transfusion”. At the end of 3 minutes, changes (Δ) in stroke volume index (SVI) and cardiac index (CI) were displayed on the NiCOM™ monitor, stating whether or not the subject was fluid responsive. The subject was then placed back in pre-PLR position. The above procedure of obtaining baseline hemodynamic values, performing the PLR, and recording ΔSVI and ΔCI was considered one PLR cycle. We performed three PLR cycles, designated as PLR 1, PLR 2, and PLR 3, each separated by a rest period of 20 to 30 minutes (Fig 1).

In the patient cohort, if the subject was found to be fluid responsive during the PLR but hemodynamically stable, data collection was continued. Upon completion of the study procedure, the primary team caring for the patient was notified of the results. If the patient was found to be fluid responsive and unstable, or the patient became hemodynamically unstable for any reason during data collection, the study procedure was aborted and the primary team was notified immediately. The study procedure was also aborted if during the data collection the patient required any new hemodynamic intervention, including titration of fluids, vasopressor and/or inotrope; or if the patient did not tolerate the PLR. Data collected from patients with the study procedure aborted were excluded from data analysis.

Subject demographic characteristics, hemodynamic parameters, Sepsis-related (or Sequential) Organ Failure Assessment (SOFA) score, laboratory values, admission diagnosis category, and the presence of vasopressor/inotrope and mechanical ventilation were recorded [14].

### Measurements of fluid responsiveness, precision and consistency

The ΔSVI after the PLR was calculated by the NiCOM™ proprietary algorithm using the peak value of SVI during the PLR challenge phase divided by the average SVI during the baseline phase. *Fluid responsiveness* was defined as ΔSVI > 10% after PLR. *Precision* was the variability in ΔSVI in response to PLR. We calculated the precision of ΔSVI over the 3 cycles of PLR’s by three methods: 1) range, 2) average deviation, and 3) standard deviation.

### Measurements of precision

Range = *x_max_* − *x_min_*

Average Deviation = $\frac{\sum_{i=1}^{n_{PLR}}\left|x_{i}-{\bar{x}}_{i}\right|}{n_{PLR}}$

Standard Deviation = $\sqrt{\frac{\sum_{i=1}^{n_{PLR}}{\left(x_{i}-{\bar{x}}_{i}\right)}^{2}}{n_{PLR}-1}}$

*x_max_* = maximum ΔSVI from 3 cycles of PLR in the same subject

*x_min_* = minimum ΔSVI from 3 cycles of PLR in the same subject

*x_i_* = ΔSVI per PLR

${\bar{x}}_{i}$ = the mean of ΔSVI computed from 3 cycles of PLR in the same subject

*n_PLR_* = 3 (number of cycles of PLR in the same subject)

*Consistency* was defined as the same fluid responsiveness determination of “Yes” (ΔSVI > 10%) or “No” (ΔSVI ≤ 10%) for all 3 cycles of PLR’s. For example, a subject was categorized to have *consistent* results if they were persistently fluid responsive or not fluid responsive for all 3 PLR’s. A subject had *inconsistent* results if any one of the 3 PLR’s resulted in a fluid responsiveness determination that was different from the other PLR’s.

### Statistical analysis

Independent sample t-test, Mann Whitney or proportion chi-square was used to test for subject differences. Repeated measures analysis of variance was used to compare hemodynamic data over the 3 cycles of PLR’s. Cohen’s κ was calculated for agreement in determining fluid responsiveness of “Yes” (ΔSVI > 10%) or “No” (ΔSVI ≤ 10%) between two PLR’s (PLR 1 vs. PLR 2, PLR 1 vs. PLR 3, and PLR 2 vs. PLR 3). Pearson’s correlation coefficient (r) was calculated to determine the linear relationship between two PLR measurements of ΔSVI. Data are reported as mean±standard deviation. Categorical data are expressed as proportions. Data were analyzed using MedCalc Statistical Software version 18.2.1 (MedCalc Software bvba, Ostend, Belgium; https://www.medcalc.org; 2016).

## Results

A total of 80 patients and 25 healthy volunteers were consented to participate in the study. Five patients were excluded: four due to a change of >1 mcg/min in norepinephrine dosage during the study procedure; and 1 withdrew their consent during the data collection period.

Final data analysis was performed on 75 patients and 25 healthy volunteers, with age 55.7±19.8 vs. 32.8±11.8 years, respectively, p < 0.01 (Table 1). Patients had statistically significant higher body mass index, heart rate, lower mean arterial pressure, higher respiratory rate, and lower SVI than volunteers (all p ≤ 0.01). SOFA score was 7.0±4.8, lactate 3.1±3.9 mmol/L, 56% were on mechanical ventilation and 32% were receiving vasopressor/inotropic support.

**Table 1:** Patient and volunteer baseline characteristics. BMI–body mass index; SOFA–sequential organ failure assessment. Data are presented as mean ± standard deviation or as count (percentage).

Characteristic		Patients N = 75	Volunteers N = 25	*p-value*
Age (years)		55.7 ± 19.8	32.8 ± 11.8	<0.01
Sex				
	Male	36 (48.0)	16 (64.0)	0.17
	Female	39 (52.0)	9 (36.0)	0.17
Race				
	White	43 (57.3)	16 (64.0)	0.54
	Black	13 (17.3)	2 (8.0)	0.27
	Asian	5 (6.7)	7 (28.0)	<0.01
	other	14 (18.7)	0 (0.0)	
BMI (kg/m^2^)		27.1 ± 6.2	22.4 ± 3.0	<0.01
Hemodynamic characteristics				
	Heart rate (beats per min)	103.0 ± 18.8	66.0 ± 11.4	<0.01
	Mean arterial pressure (mm Hg)	76.0 ± 15.4	84.0 ± 13.3	0.01
	Respiratory rate (breaths per min)	21.0 ± 6.1	15.0 ± 3.0	<0.01
	Stroke volume index (mL/min/m^2^)	33.5 ± 9.2	55.7 ± 10.3	<0.01
	Cardiac index (L/min/m^2^)	3.3 ± 0.9	3.5 ± 0.6	0.08
	Total peripheral resistance (dyne*sec/cm^-5^)	1106.0 ± 499.9	1080.4 ± 398.5	0.79
Laboratories				
	Creatinine (mg/dL)	1.2 ±1.2	-	-
	Lactate (mMol/L)	3.1 ± 3.9	-	-
SOFA		7.0 ± 4.8	-	-
Hospital day (days)		5.0 ± 5.6	-	-
Admission diagnosis category				
	Sepsis	44 (58.7)	-	-
	Cardiac	11 (14.7)	-	-
	Respiratory	12 (16.0)	-	-
	Malignancy	2 (2.7)	-	-
	Metabolic	3 (4.0)	-	-
	Hematologic	3 (4.0)	-	-
	Renal	1 (1.3)	-	-
	Neurology	1 (1.3)	-	-
Presumed etiology of shock				
	Septic / distributive	41 (54.7)	-	-
	Cardiogenic	16 (21.3)	-	-
	Hypovolemic	3 (4.0)	-	-
	Obstructive	0 (0.0)	-	-
	Unknown	15 (20.0)	-	-
Reason for enrollment				
	Hypotension	26 (34.6)	-	-
	Tachycardia	59 (78.7)	-	-
	Skin mottling	3 (4.0)	-	-
	Lactate > 2.0mm/L	32 (42.7)	-	-
	Oliguria	12 (16.0)	-	-
	Vasopressor / inotropic support	24 (32.0)	-	-
Mechanical ventilation		42 (56.0)	-	-

**Fig 2:**
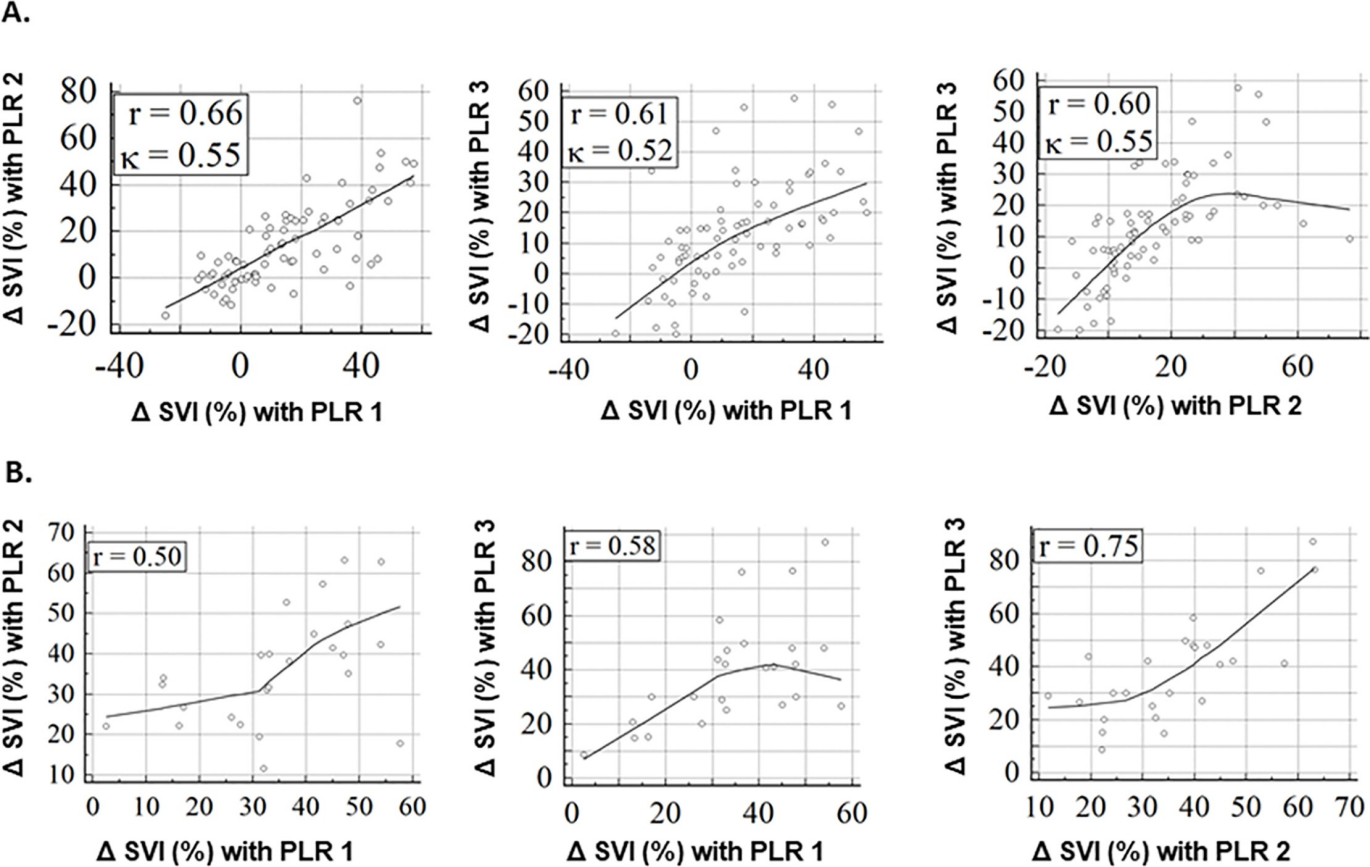
Correlation and agreement between cycles of passive leg raising (PLR) maneuvers. A. Patients. B. Volunteers. r–Pearson’s correlation coefficients. K–Cohen’s kappa for agreement in determining fluid responsiveness of “Yes” (ΔSVI > 10%) or “No” (ΔSVI ≤ 10%) between two PLR maneuvers. K was not calculated for volunteers since the consistency in determining fluid responsiveness over all three PLR maneuvers was 96.0% (Table 3).

There was no statistical difference in ΔSVI or ΔCI over the 3 cycles of PLR’s in both patients and volunteers (Table 2). For patients, the linear relationship between ΔSVI’s in each pair of PLR’s (PLR 1 vs. PLR 2, PLR 1 vs. PLR 3, and PLR 2 vs. PLR 3) showed Pearson’s correlation r = 0.66, 0.61 and 0.60, respectively (Fig 2A). For volunteers, r = 0.50, 0.58 and 0.75, respectively (Fig 2B). The agreement in determining fluid responsiveness between two PLR’s in patients showed K = 0.55, 0.52 and 0.55, respectively.

**Table 2:** Change in hemodynamic measurement with three cycles of passive leg raise (PLR) maneuvers. SVI–stroke volume index; CI–cardiac index; ΔSVI–change in stroke volume index after the PLR maneuver; ΔCI–change in cardiac index after the PLR maneuver. Data are represented as mean ± standard deviation.

Patients N = 75	PLR 1		PLR 2		PLR 3		*p-value*
	Baseline	After PLR	Baseline	After PLR	Baseline	After PLR	
SVI (mL/min/m^2^)	33.0 ± 9.8	38 ± 11.5	33.4 ± 8.4	37.6 ± 11.2	34.2 ± 10.8	37.8 ± 12.1	-
CI (mL/min/m^2^)	3.3 ± 0.9	3.7 ± 1.0	3.3 ± 0.8	3.8 ± 1.0	3.4 ± 1.0	3.7 ± 1.0	-
ΔSVI (%)	-	14.5 ± 19.7	-	14.7 ± 18.5	-	13.2 ± 16.9	0.59
ΔCI (%)	-	14.7 ± 19.4	-	14.6 ± 19.2	-	12.7 ± 17.4	0.59
**Volunteers** **N = 25**							
SVI (mL/min/m^2^)	55.7 ± 10.4	74.4 ± 13.2	53.0 ± 9.7	71.9 ± 13.7	51.6 ± 10.6	71.2 ± 14.6	-
CI (mL/min/m^2^)	3.5 ± 0.6	4.6 ± 0.8	3.3 ± 0.5	4.3 ± 0.8	3.1 ± 0.6	4.26 ± 0.9	-
ΔSVI (%)	-	34.7 ± 14.3	-	36.1 ± 13.8	-	39.2 ± 19.9	0.68
ΔCI (%)	-	30.6 ± 12.5	-	32.2 ± 13.7	-	35.8 ± 17.3	0.69

The precision of ΔSVI over 3 cycles of PLR’s was determined by *range*, *average deviation* and *standard deviation* (Table 3). Range was 17.2±13.3% and 17.4±10.3%, average deviation was 6.5±4.0% and 6.6±3.8%, and standard deviation was 9.0±5.2% and 9.0±6.7%, in patients and volunteers, respectively. There was no statistical difference in the precision measurements between patients and volunteers.

**Table 3:** Precision and consistency of passive leg raise in determining fluid responsiveness. *Precision* is described by the range, average deviation, and standard deviation of the change in stroke volume index (ΔSVI) over 3 cycles of passive leg raising (PLR) maneuver. *Consistency* was defined as the same fluid responsiveness determination of “Yes” (ΔSVI > 10%) or “No” (ΔSVI ≤ 10%) over the 3 cycles of PLR maneuver. For example, a subject was categorized to have *consistent* results if they were persistently fluid responsive or not fluid responsive for all 3 cycles of PLR maneuver. A subject had *inconsistent* results if any one of the 3 cycles of PLR maneuver resulted in a fluid responsiveness determination that was different from the other PLR’s. Data are represented as mean ± standard deviation or as count (percentage).

Precision	Patients N = 75	Volunteers N = 25	*p-value*
Range of ΔSVI (%)	17.2 ± 13.3	17.4 ± 10.3	0.94
Average deviation of ΔSVI (%)	6.5 ± 4.0	6.6 ± 3.8	0.91
Standard deviation of ΔSVI (%)	9.0 ± 5.2	9.0 ± 6.7	0.15
**Consistency**			
Consistent determination of fluid responsiveness	49 (65.3)	24 (96.0)	<0.01
Consistently fluid responsive with ΔSVI > 10%	24 (32.0)	24 (96.0)	<0.01

Forty-nine (65.3%) patients had consistent determination of fluid responsiveness for all 3 PLR’s (Table 3). Among these, twenty-four (49.0%) patients were consistently fluid responsive and 25 (51.0%) were consistently *not* fluid responsive. Among volunteers, twenty-four (96.0%) had consistent determination of fluid responsiveness, with all those being fluid responsive (ΔSVI > 10%).

## Discussion

We showed that the percent change in SVI measured by the NiCOM™ after a PLR has a precision of approximately ±9% (standard deviation) in both critically ill patients and healthy volunteers. While the *accuracy* of the PLR in predicting fluid responsiveness has been studied, our data suggest that the *precision* can change clinical decisions regarding fluid management when using this technology [13]. For example, a ΔSVI of 15% after PLR may be interpreted as 15±9%. In this scenario, should fluids be administered or should the PLR be repeated? If we repeat the PLR, our data showed that after 3 PLR’s, ΔSVI would be consistent in 65% of patients and 96% of healthy volunteers using the cutoff of 10%.

Previous studies have shown that CO has a variation of 4.4 to 10.1% [15, 16]. However, repeated measures analysis of variance showed no significant differences between CO measurements [15]. Similarly, in our study, repeated measures analysis of variance showed no significant differences in ΔSVI amongst the 3 cycles of PLR’s. Yet the variability (standard deviation) of 9% in ΔSVI has significant clinical ramification when a cutoff of 10% is used to determine fluid responsiveness by the NiCOM™.

In addition to spontaneous variation in CO measurements as explanation for our results, we used a bioreactance-based technology. While a number of studies have shown bioreactance to be a viable technology in determining fluid responsiveness coupled with the PLR, Kupersztych-Hagege et al. showed it to be unreliable when compared to thermodilution in measuring CI, with an error up to 82% and an area under the receiver operating characteristics curve not different from 0.5 for the ability of PLR induced ΔSVI to predict fluid responsiveness [17]. In a validation study by Squara et al., continuous CO measured by the NiCOM™ technology was compared to the PA catheter. During periods of stable CO, the precision (defined as 2 standard deviation / mean) of CO measured by NiCOM™ was 12±7% [12]. Stetz et al. had previously shown that a difference of 12–15% in CO measurements may suggest clinical significance [18]. Thus, the variability in ΔSVI after serial PLR’s observed in our study may be contributed by inaccurate technology and/or clinically significant spontaneous changes in SV.

Lamia et al. further examined the accuracies of continuous CO measurements by various technologies incorporating arterial pulse contour analysis, bioreactance and thermodilution [19]. They found that compared to the pooled CO measured by all devices, the percentage error for CO was 34, 34, 49, 28 and 37% for LiDCO^TM^ (LiDCO Ltd, London, UK), FloTrac^TM^ (Edwards Life Science, Irvine, CA, USA), NiCOM^TM^, PA catheter and PiCCO^TM^ (Pulsion Ltd, Munich Germany), respectively. Thus, we posit that the variability in ΔSVI observed with NiCOM^TM^ in our study will likely be similar with other technologies.

Our study showed that healthy volunteers were more fluid responsive than critically ill patients, with 96% of the volunteers being consistently fluid responsive with ΔSVI > 10%. Similarly, Miller et al. examined 40 healthy volunteers by performing the PLR followed by 500 mL intravenous bolus of 0.9% normal saline [20]. They showed that 90% of the subjects were fluid responsive. Kumar et al. also examined the hemodynamic responses to volume infusion in normal subjects using the PA catheter, and showed that 3 L of normal saline resulted in 30% increased CI and 23% increased SVI [21]. In our study, the hospital day at the time of enrollment was 5.0±5.6 days. Thus, many patients have already received some amount of fluid resuscitation, leading to less fluid responsiveness compared to volunteers.

We performed an observational study without a reference hemodynamic monitoring technology, such as the PA catheter, to compare the NiCOM^TM^ against. We also did not confirm fluid responsiveness with administration of a fluid bolus. Recent systematic reviews already concluded that the PLR was the most accurate measure of fluid responsiveness compared to other modalities [5, 13]. Our aim was to determine the precision or repeatability of ΔSVI after PLR, and not its accuracy. Furthemore, fluid administration in between PLR’s will change the patient’s location on the Starling curve and invalidate the purpose of our study.

We did not have a priori estimate of the precision of ΔSVI after the PLR in the general population. Therefore, we could not perform a sample size calculation taking into consideration statistical vs. clinical significance. However, based on previous studies examining the accuracy of PLR, our study team arbitrarily agreed to enroll 75 critically ill patients and 25 healthy volunteers [13].

Previous studies examining the variability of CO have taken measurements at intervals of seconds to minutes [15, 16]. We used a pragmatic interval of 20–30 minutes allowing for data collection and subject repositioning in between consecutive PLR’s. This longer interval may result in physiologic changes in CO and ΔSVI. However, we observed that SVI and CI did in fact return to baseline values during this interval. Additionally, it is not uncommon in standard practice for volume assessment to be repeated at 20–30 minute intervals, rather than seconds or a few minutes, after an intervention.

## Conclusion

In conclusion, our study showed the precision of determining ΔSVI by the NiCOM™ after the PLR is at ±9% in both critically ill patients and volunteers; however, consistency is significantly higher in volunteers. Although not tested, knowledge of precision and consistency of the PLR in predicting fluid responsiveness may impact clinical decision-making. The ΔSVI cutoff of 10% may need further clarification as 10±9%. With this level of variability, fluid responsiveness could perhaps be redefined as graded levels of responsiveness rather than as a binary prompt when utilizing non-invasive hemodynamic monitoring technologies. For example, ΔSVI < 5% may suggest “not fluid responsive”, ΔSVI 5–15% suggests “possible fluid responsive”, and ΔSVI > 15% “definitely fluid responsive”. Further studies are thus needed to examine how precision of the PLR and such proposed modified definition of fluid responsiveness can be incorporated in fluid resuscitation protocols.

## Supporting information

S1 File Data set used to reach the conclusion drawn in the manuscript. (XLSX)
